# The Contribution of Attachment Styles and Reassurance Seeking to Trust in Romantic Couples

**DOI:** 10.5964/ejop.3059

**Published:** 2022-02-25

**Authors:** Lyndsay Elizabeth Evraire, David John Andrew Dozois, Jesse Lee Wilde

**Affiliations:** 1 Department of Psychology, University of Western Ontario, London, ON, Canada; Ohio State University Wexner Medical Center, Columbus, OH, USA

**Keywords:** reassurance seeking, attachment styles, trust, daily diary

## Abstract

The current daily diary study examined the moderating impact of attachment style on the association between excessive reassurance seeking (ERS) behavior and trust in romantic dyads. A sample of 110 heterosexual couples completed measures of attachment, ERS, and relationship trust. In line with prior research, an anxious attachment style was associated with higher daily ERS, and an avoidant attachment style with lower daily ERS. Lower levels of trust were also associated with greater daily ERS. Moreover, analyses remained significant while controlling for symptoms of depression. This study extended the literature by demonstrating that for women with an anxious attachment style, and men with an avoidant attachment style, ERS was related to lower next day trust. In contrast, the partners of men with an avoidant attachment style, who also engaged in ERS, reported higher levels of next day trust. This study was also the first to examine how individual attachment styles influenced the perception of, and reactions to, ERS. Women with an anxious attachment style liked when their male partners engaged in ERS, as illustrated by higher levels of reported trust. These results support the idea that attachment styles play an important role in determining whether or not ERS leads to negative interpersonal consequences. They also suggest that it is the combination of relationship insecurities and ERS that leads to negative interpersonal consequences.

Excessive reassurance seeking (ERS) is defined as “the relatively stable tendency to excessively and persistently seek assurances from others that one is lovable and worthy, regardless of whether such assurance has already been provided” (Joiner, Metalsky, Katz, & Beach, 1999, p. 270). In recent years, studies examining the causes and consequences of ERS in interpersonal relationships have proliferated. Research has repeatedly shown associations between ERS and a variety of personal and interpersonal negative outcomes, including reduced self-worth, interpersonal rejection, deteriorating relationship quality, increased symptoms of depression, anxiety, and deteriorating mental health more broadly (e.g., Cougle et al., 2012; Evraire & Dozois, 2011, 2014; Lemay & Cannon, 2012; Lord, Harkness, Suvak, & Stewart, 2020; Starr, 2015; Starr & Davila, 2008; Stewart & Harkness, 2017). As Coyne’s original model of ERS was developed as a theory of depression, most of the extant literature has examined ERS and depression as a primary outcome of interest. However, more research is needed to understand the interpersonal consequences of ERS on romantic relationship functioning. Additionally, while Coyne’s interpersonal model has greatly advanced our understanding of ERS behavior in depression, the original theory needs to be further refined to incorporate recent research that has examined various causes and consequences of ERS (i.e., Evraire & Dozois, 2011, 2014; Lord et al., 2020; Shaver, Schachner, & Mikulincer, 2005; Stewart & Harkness, 2017).

In his interpersonal theory of depression, Coyne (1976) asserted that, in response to their symptoms of guilt and low self-worth, individuals with mild depression seek reassurance from close others to test the security of their relationships. Although these bids for reassurance are initially met with the requested support from others, continued reassurance-seeking behavior causes close others to become frustrated and ultimately reject the individual with depression (Joiner, Alfano, & Metalsky, 1992). Recent research suggests that individuals may engage in ERS not because of depression *per se*, but as a function of important individual differences (such as attachment styles or core beliefs) that reflect high levels of concern surrounding relationships (e.g., Evraire & Dozois, 2014). Moreover, research in the ERS literature has begun to examine how individual difference variables may moderate the association between ERS and negative personal and interpersonal outcomes. That is, it may not be the behavior of ERS per se, but rather characteristics of the *individual* or the *relationship* in combination with ERS that are associated with negative consequences (e.g., Evraire & Dozois, 2011, 2014; Lord et al., 2020; Stewart & Harkness, 2017). Indeed, recent studies suggest that ERS is not always deterimental and may have positive effects for individuals and relationships under certain circumstances (e.g., Abe & Nakashima, 2020; Fowler & Gasiorek, 2017; Girme, Molloy, & Overall, 2016). Clearly, a more nuanced understanding of factors contributing to an individual’s engagement in, and negative responses to, ERS is needed.

## Attachment Style as a Predictor of Engaging in Excessive Reassurance Seeking Behavior

A growing body of research has implicated the role of attachment style in ERS behavior (Evraire & Dozois, 2011, 2014; Evraire, Ludmer, & Dozois, 2014; Shaver et al., 2005). According to attachment theory, infants learn to self-soothe and self-reassure through the development of a secure attachment to caregivers early in life. Conversely, when an infant’s caregiver is inconsistent in responding to the child’s needs, an insecure attachment is said to develop, and the child learns to seek assurances externally rather than developing the ability to self-soothe (Bowlby, 1980). These early interactions between an infant and their caregiver lead to the development of internal working models (IWMs) about the self, others, and their interrelationships. IWMs of attachment influence not only the way individuals relate to others, but also their attributions, perceptions, and emotional understanding of these relationships (Moran, Bailey, & DeOliveira, 2008). Positive models of self (as worthy of love and nurturance) and others (as responsive and trustworthy) reflect attachment security, whereas negative models of self (as unworthy of love and nurturance) and/or others (as unresponsive and untrustworthy) reflect insecurity (Bretherton & Munholland, 2008). In adults, attachment style is conceptualized along two dimensions: avoidance, which involves feeling discomfort in close relationships, and anxiety, which involves worrying about the availability of others to meet attachment needs and one’s self-worth in relation to others (Fraley, 2019). Attachment avoidance is thought to be associated with positive IWMs of self and negative IWMs of other. Conversely, attachment anxiety is thought to be associated with negative IWMs of self and positive IWMs of others (Holmes & Johnson, 2009).

Research supports the notion that adults with an avoidant attachment style minimize the expression of negative emotions and use *deactivating* strategies (such as avoiding proximity to attachment figures) to manage distress, whereas anxiously attached individuals tend to maximize the expression of negative emotions and use *hyperactivating* strategies (such as seeking proximity to attachment figures) to regulate distress (Malik, Wells, & Wittkowski, 2015; Mikulincer & Shaver, 2016). From an attachment framework, then, ERS behaviour can be conceptualized as a strategy that individuals may use to manage distress and assuage any doubts about their lovability, worthiness (i.e., self-esteem), future prospects, and safety (i.e., anxiety). Indeed, research supports the notion of ERS as a coping strategy in response to interpersonal threats, stress, or anxiety (Evraire et al., 2014; Cougle et al., 2012; Joiner, Katz, & Lew, 1999; Parrish & Radomsky, 2011).

Given the different strategies for managing distress that are characteristic of anxious and avoidant individuals, it would follow that individuals with anxiety are more inclined to engage in ERS (a hyperactivating strategy) than individuals engage in avoidance (who favour deactivating strategies). In line with this, research has repeatedly shown an association between attachment anxiety and higher levels of overall and daily ERS (Abela et al., 2005; Davila, 2001; Evraire & Dozois, 2014; Evraire et al., 2014; Katz, Petracca, & Rabinowitz, 2009; Shaver et al., 2005). As individuals with anxious attachment hold positive IWMs of others in addition to low self-esteem and a fear of abandonment, they are more likely to rely on reassurance from others to confirm their self-worth and relationship security (Mikulincer & Shaver, 2016). But, these anxious individuals may continue to engage in ERS as a result of inconsistent caregiving that taught them to distrust cognitive information when trying to predict an attachment figure’s behavior (Mikulincer & Shaver, 2016). Conversely, avoidant individuals may be less inclined to seek reassurance from others during times of distress as a result of their negative IWMs of others.

## Attachment Style as a Predictor of the Negative Consequences of Excessive Reassurance Seeking Behavior

Not only is attachment style an important contributor to an individual’s tendency to engage in ERS behaviour, it may also influence the degree to which ERS behaviour has negative implications for the individual engaging in ERS. For example, Shaver and colleagues (2005) found that for women high in attachment anxiety, reassurance-seeking on a given day was associated with greater self-reported negative mood the next day. For non-anxious women, however, reassurance-seeking on a given day led to greater reported positive mood the next day. Similarly, Evraire and Dozois (2014) found that for individuals with greater fears of abandonment and relational insecurity, ERS was associated with higher levels of depressive symptomatology over a 6-week period. However, for individuals high in avoidant attachment, there was no association between ERS and subsequent symptoms of depression. These findings suggest that, in response to their perception of close others as unreliable and their fear of abandonment, individuals with doubts about relationship security seek reassurance in a way that is likely aversive to others and detrimental to their own psychological well-being. However, for avoidant individuals who typically distance themselves from the support of close others, ERS does not significantly impact their own levels of depressive symptoms. In this way, ERS behaviour may be more or less detrimental to an individual’s well-being depending on their attachment style. More research is needed to understand whether attachment style also moderates the effecs of an individual’s ERS on relationship outcomes.

An individual’s attachment style may also have important implications for how they react to a romantic partner’s ERS behavior. That is, Partner A’s attachment style may influence how they outwardly respond to Partner B’s ERS. For example, Simpson, Rholes, and Nelligan (1992) found that avoidant men offered less reassurance, supportive comments, and emotional support to their female partners following a stressor than did secure men. Additionally, Partner A’s own attachment style may also moderate the effects of Partner B’s ERS on Partner A’s feelings about the relationship. For instance, Shaver and colleagues (2005) found that avoidant women experience a partner’s ERS behaviors as particularly aversive and as having a negative influence on self-reported relationship quality. Knowing that individuals with an avoidant attachment style down-regulate attachment feelings and behaviors by distancing themselves from their partners, it makes intuitive sense that having a partner constantly asking for reassurance would be aversive to avoidant individuals, given that seeking reassurance is incongruent with their own strategy of coping with distress.

Further, the attachment style of an individual engaging in ERS may influence their partner’s response to ERS. That is, Partner A’s attachment style may infuence Partner B’s reactions to Partner A’s ERS behaviour. In particular, past research suggests that ERS behaviour may be particularly aversive if is it coming from an individual who is higher in attachment anxiety (Evraire & Dozois, 2011, 2014; Lord et al., 2020). For example, Stewart and Harkness (2017) found that in women with higher reported fear of abandonment and relationship insecurity, ERS behaviour predicted reduced relationship quality reported by their partner. It is possible that these individuals convey a greater sense of urgency and desperation that is particularly offputting to partners on the receiving end of this behaviour. Girme et al. (2016) reported a similar pattern of findings for attachment anxiety, and also found that ERS from an avoidant partner appeared to “repair the lack of closeness” that individuals with avoidant partners may often feel. In other words, ERS may actually have positive effects for the romantic partners of avoidant individuals. Taken together, the findings suggest that attachment style may moderate the impact of ERS on personal and interpersonal well-being. Moreover, there appears to be both within-partner and cross-partner effects of attachment, suggesting that the use of sophisticated dyadic analyses is key to elucidating these complex interpersonal phenomena.

## Trust, Attachment, and Excessive Reassurance Seeking

Evraire and Dozois’ (2011) integrated model of reassurance-seeking implicates trust as an important construct to consider when conceptualizing potential origins and consequences of ERS. Within the context of romantic relationships, trust has been conceptualized in the literature as the confidence that an individual’s partner will be concerned about and responsive to their needs, desires, and goals, along with faith in the future of the relationship (Mikulincer, 1998). Difficulty trusting others may be associated with attachment insecurity (Campbell & Stanton, 2019; Fitzpatrick & Lafontaine, 2017). For instance, despite their solicitation of and openness to feedback, individuals with an anxious attachment style often do not believe the reassurance they receive from close others and thus continue to engage in ERS (Evraire & Dozois, 2014). In this way, low levels of trust may represent an important contributor to ERS in that it drives individuals to doubt a partner’s regard for them, and thereby continue to seek reassurance. Mistrust may also represent an important consequence or byproduct of ERS, as repeated attempts to obtain reassurance that are met with frustration and rejection from a partner may begin to erode trust over time. Surprisingly, no research to date has examined the association between relationship trust and reassurance-seeking behaviour. Given that trust is a critical component of dyadic adjustment and relationship well being (Campbell & Stanton, 2019), a more thorough understanding of the dynamic interplay among trust, attachment, and ERS is needed in the literature. As such, the current study aims to provide the first empirical examination of these associations as they unfold overtime between romantic partners.

## Limitations of the ERS Literature and Need for the Current Study

Interpersonal schemas reflecting insecurity in relationships, particularly a fear of rejection or abandonment, have demonstrated incremental predictive power for ERS over and above the influence of depression (Evraire & Dozois, 2011, 2014; Evraire et al., 2014). As reviewed previously, recent research suggests that individuals may engage in ERS, not because of depression per se, but as a function of certain individual difference variables (such as attachment styles or high levels of concern surrounding relationships; Evraire & Dozois, 2014). As such, the details of Coyne’s model may need to be refined to incorporate these findings. In the current study, symptoms of depression were included as a control variable throughout analyses to determine whether attachment anxiety predicts aspects of the ERS model above and beyond depression. Should the proposed study replicate the findings of Evraire and Dozois (2014), they would have serious implications for the revision of the ERS model as originally proposed by Coyne.

In addition to the abovementioned conceptual limitations in the ERS literature, several methodological limitations must be addressed. One aspect of the ERS literature that is frequently criticized involves the almost complete lack of methodological diversity across studies. The most recent meta-analysis found that over two-thirds of ERS studies used college-aged samples (between the ages of 18 and 22 years), with only seven examining ERS in children, and five testing ERS in post-college aged adults (Starr & Davila, 2008). The failure to examine ERS across the lifespan is an important shortcoming given that the nature of interpersonal relationships and the social acceptability of ERS fluctuate substantially with age (Starr & Davila, 2008). Moreover, an additional limitation concerns the lack of prospective studies that examine the contribution of ERS to relationship outcomes. Furthermore, some aspects of the ERS model are predicated on the notion that these processes unfold over days rather than months (e.g., that reassurance seeking on 1 day leads to negative consequences the next); yet, to our knowledge, only two studies have examined reassurance seeking behaviours at the daily level of analysis (Shaver et al., 2005; Starr, 2015). Moreover, all of the prospective studies conducted to date have used an undergraduate sample. Finally, given that examinations of cross-partner effects of ERS are critical to understanding the interpersonal consequences of this behaviour, dyadic analyses are of the utmost importance. Dyadic approaches afford the opportunity to understand complex and dynamic interpersonal processes as they unfold within a romantic relationship context. As such, the current study provides an important contribution to the literature through the use of a longitudinal, daily diary design and a non-undergraduate sample of romantic dyads recruited from the community.

## Objectives and Hypotheses

The current study aims to address several limitations of the ERS literature to replicate previous findings and provide an original contribution to the literature by elucidating a more comprehensive understanding of the daily dynamics and ramifications of ERS in romantic relationships. The research objectives and hypotheses for the current study are outlined below.

### Objective 1: To Examine the Contribution of Attachment Style and Trust to Daily ERS

The first objective of this study was to replicate the cross-sectional association among attachment styles and ERS over a 14-day period in a community sample of romantic couples, and to provide the first examination of the association between ERS and trust.

***H1:*** Higher attachment anxiety was expected to be associated with greater daily ERS.

***H2:*** Higher attachment avoidance was expected to be associated with lower daily ERS.

***H3:*** Lower levels of reported relationship trust was expected to be associated with increased ERS.

### Objective 2: To Examine the Moderating Properties of Attachment Style on the Relationship Between Daily ERS and Next Day Trust

***H4:*** In line with previous research, individuals high in attachment anxiety, who also engaged in ERS, were expected to experience lower next day relationship trust. Similarly, individuals higher in attachment avoidance were expected to experience lower next day trust as well.

### Objective 3: To Examine the Moderating Properties of Actor Attachment Style on the Relationship Between Actor Daily ERS and Partner Next Day Trust

The third objective was to examine how an individual’s attachment style moderated the impact of their own ERS behavior on a partner’s next day trust.

***H5:*** Given that past research suggests anxiously attached individuals may seek reassurance in a way that is likely aversive to others and detrimental to their psychological well-being, it was predicted that individuals with an anxious attachment style would negatively impact their partner’s reported level of trust when they engage in ERS.

### Objective 4: To Examine the Moderating Properties of Actor Attachment Style on the Relationship Between Partner Daily ERS and Actor Next Day Trust

The final objective was to examine how an individual’s attachment style influenced how they reacted to receiving ERS from their partner in terms of trust.

***H6:*** A partner’s ERS was hypothesized to lead to low trust in individuals higher in attachment avoidance.

## Method

### Participants

One-hundred and ten heterosexual couples were recruited from London, Ontario, Canada. The age of participants ranged from 16^1^1The sample included only one adolescent couple under the age of 18 years. All analyses reported below were re-run with and without the adolescent couple, and results did not differ significantly.
to 68, with a mean age of 32.45 years (*SD* = 10.32). Couples had been together for periods ranging from 2 months to 46 years (*M* = 7.95 years, *SD* = 7.86). Of the 220 participants, reported race was 82.9% Caucasian, 3.6% Asian, 1.4% African Canadian, 3.6% First Nations or Native Canadian, 1.8% Hispanic, and 6.7% other. The majority of participants were employed outside of the home (82.9%) and, overall, the sample was highly educated (highest level of education: 6.8% completed some highschool; 11.3% completed highschool or General Education Development (GED) testing; 55.8% completing/completed college or university; 26.1% either completing/completed graduate or profesional school). Couples were required to be living together at the time of participation and had been cohabitating anywhere from 1 month to 45 years (*M* = 6.44 years, *SD* = 7.71). Although not necessary for participation, roughly half (55%) of the couples were married. Overall, individuals in this sample reported relatively high levels of perceived relationship quality (*M* = 109.1, *SD* = 14.7, possible scores ranging from 1 to 126).

### Laboratory Measures

#### Beck Depression Inventory, Second Edition

The Beck Depression Inventory, Second Edition (BDI-II; Beck, Steer, & Brown, 1996) is a 21-item questionnaire that assesses the presence and severity of unipolar depressive symptomatology. Each item is rated on a 4-point scale from 0 (*indicating a lack of depressive symptomatology*) to 3 (*indicating high depressive symptomatology*) with summary scores ranging from 0 to 63. Considerable psychometric evidence supports the internal reliability, concurrent, and discriminant validity of this questionnaire as a measure of depression in both clinical and undergraduate samples (Beck & Steer, 1987; Dozois, Dobson, & Ahnberg, 1998). The internal consistency (Cronbach’s alpha) of the BDI-II in this sample was 0.91 in women and 0.89 in men.

#### Depressive Interpersonal Relationships Inventory-Reassurance Seeking Subscale

The Depressive Interpersonal Relationships Inventory-Reassurance Seeking Subscale (DIRI-RS; Joiner, et al., 1992) is a 4-item self-report questionnaire designed to measure an individual’s tendency to engage in reassurance seeking (e.g., “Do you find yourself often asking the people you feel close to how they truly feel about you?”), and their partner’s reactions to such reassurance seeking (e.g., “Do the people you feel close to sometimes get fed up with you seeking reassurance from them about whether they really care about you?”). Participants answer the questions based on their current relationships on a 7-point scale from 1 (*not at all*) to 7 (*very much*). An average score was calculated with scores ranging from 1 to 7. Joiner and Metalsky (2001) supported the construct and criterion validity of the DIRI-RS along with its use as a cohesive and replicable measure of reassurance-seeking distinct from general dependency, doubt in others’ sincerity, and dependence on close others. The DIRI-RS demonstrates high internal consistency (Joiner et al., 1992). Coefficient alpha in the present sample was 0.90 for women and 0.83 for men.

#### Experiences in Close Relationships-Revised

The Experiences in Close Relationships-Revised (ECR-R; Fraley, Waller, & Brennan, 2000) is a revised version of Brennan, Clark, and Shaver’s (1998) Experiences in Close Relationships (ECR) questionnaire. This 36-item questionnaire is designed to assess individual differences with respect to attachment anxiety (the extent to which people are insecure about their partner’s availability and responsiveness) and attachment-related avoidance (the extent to which individuals are uncomfortable being close to others). Participants rate each item on a 7-point scale from 1 (*disagree strongly*) to 7 (*agree strongly*) based on experiences in their current relationship. Attachment anxiety scores were created by averaging responses across the anxiety dimension items and ranged from 1 to 7. Attachment avoidance scores were created by averaging responses across the avoidance dimension items and ranged from 1 to 7. The internal consistency reliability of the ECR-R is excellent (e.g., α ≥ 0.90). Coefficient alpha for the current sample on attachment anxiety was 0.93 for women and 0.91 for men, and for attachment avoidance 0.91 for women, and 0.92 for men.

#### Perceived Relationship Quality Component Inventory—Trust Scale

The Perceived Relationship Quality Component Inventory Trust Scale (PRQC; Fletcher, Simpson, & Thomas, 2000) is a 3-item subscale of the PRQC designed to asses an individual’s perceived level of trust in a current romantic relationship. Each item is rated on a 7-point scale from 1 (*not at all*) to 7 (*extremely*). Responses were averaged across each component with scores ranging from 1 to 7 and summed to form a global index of relationship quality ranging from 1 to 126, with higher scores indicating greater perceived relationship quality. The trust scale composite from the PRQC was used as the measure of trust in this study. The psychometric properties of this measure are strong, demonstrating good reliability and high face validity as a measure of specific domains of relationship quality (Fletcher et al., 2000). For the current sample, coefficient alpha for trust was 0.88 for women and 0.87 for men.

### Diary Measures

#### Daily Reassurance Seeking

As the DIRI-RS is the most widely used index of ERS in the literature, an abbreviated version of this measure was used to assess daily reassurance-seeking behavior. Two items were taken directly from the DIRI-RS and used for the daily diary measure: (a) how much did you seek reassurance from your partner today about whether he or she really cares about you? and (b) did your partner become irritated or get fed up with you today for seeking reassurance about whether he or she really cares about you? Participants answered the questions based on their current relationship on a 7-point scale from 1 (*not at all*) to 7 (*very much*). An individual’s ERS was calculated by averaging items (a) and (b) with scores ranging from 1 to 7. It is important to note that, although the psychometric properties of this modified version of the DIRI-RS remain unclear, abbreviated versions or single-item measures are standard practice in daily diary studies, largely due to the need to minimize participant burden and retain participation. In line with this, the two other daily diary studies examining ERS used similarly abbreviated versions of the DIRI-RS (Shaver et al., 2005; Starr, 2015). Furthermore, in the current study, there was a significant association between global reports of ERS as measured by the DIRI-RS, and averaged scores of daily reassurance seeking (*r* = .19, *p* < .01). In the present sample, coefficient alpha was 0.79 for women and 0.87 for men.

#### Daily Trust

A single item from the PRQC—Trust scale (Fletcher et al., 2000) was used to assess daily relationship trust. On a 7-point scale ranging from 1 (*not at all*) to 7 (*extremely*) participants were asked to rate how much they trusted their partner that day. There was a significant association between global reports of trust (as measured by the PRQC subscale), and averaged scores of daily trust (as measured by this single item; *r* = .72, *p* < .01).

#### Daily Negative Mood

The Negative Affect Scale from the Positive and Negative Affect Scale (PANAS; Watson, Clark, & Tellegen, 1988) was used. The Negative Affect Scale is a 10-item questionnaire that assesses both negative affect (10 items each). Each item is rated on a 5-point scale from 1 (*very slightly or not at all*) to 5 (*extremely*) and inquires about the extent to which the individual currently feels the emotion (e.g., “Indicate the extent to which you currently feel distressed”). A total negative/dysphoric mood score was computed by summing the 10 negative adjectives with scores ranging from 1 to 50. Initial studies in development of the PANAS showed that the mood scales are stable at appropriate levels over a 2-month time period, highly internally consistent, and largely uncorrelated (Crawford & Henry, 2004; Watson et al., 1988). Coefficient alpha for negative mood in the present sample was 0.86 for women and 0.90 for men.

### Procedure

A convenience sample of romantic dyads was recruited from the general community. Participants were recruited through the use of fliers posted in local businesses in London, Ontario, Canada area (e.g., grocery stores) and through online advertisements on Kijiji and in local hospitals. Interested participants contacted the lab, and individuals who met criteria for participation in the study (i.e., they were living with a romantic heterosexual partner), were scheduled for a lab visit with their partners. All interested participants who contacted the lab were eligible for participation; as such, no couples were excluded based on eligibilty criteria. All study procedures, and relevant ethical considerations, were approved by the University’s research ethics board.

#### Phase 1

This study had two phases: an in-lab phase (Phase 1) and a daily diary phase (Phase 2). Upon entry into the study, informed consent was obtained from all individuals before beginning any study measures. Couples were run one at a time for Phase 1. In Phase 1, each member of the couple completed a package of questionnaires (including the BDI-II, DIRI-RS, ECR-R, and PRQCI-Trust). Upon completion of the in-lab study, couples were asked to participate in an additional 14-day diary study in which they were asked to fill out daily questionnaires online. Participants were compensated monetarily ($50/couple) for their participation in the study regardless of whether or not they participated in Phase 2.

#### Phase 2

All couples agreed to participate in Phase 2 and so were given instructions on how to complete the questionnaire online at the end of each day for 14 days (separate from their partner) to assess their mood, trust, and ERS. Each couple watched a demonstration by the research assistant on how to open the link to the online questionnaire from an email which would be sent to them daily. Throughout the study, daily participation was monitored and participants who failed to complete a day of the diary were contacted (by phone or email) to ensure they were not having difficulties with the online questionnaire and to encourage active participation. Participants were also encouraged to contact the lab at any time with questions they had. For each day that participants completed the daily measures, their name was entered into a draw that would be completed at the end of the study for a chance to win one of 4 iPads. Furthermore, upon completion of the 14-day daily diary phase, couples were compensated monetarily for each day they completed the online diary ($3/day per individual). On average, participants completed 12.78 diary entries.

### Data Analytic Approach

Diary data were hierarchically nested in a two-level crossed design. Participants completed questionnaires each day for 14 consecutive days. The data have three levels of analysis: the dyad, the partners within the dyad, and the observations within the persons. However, the within-person observations are crossed rather than nested, so that the first day of the diary study for the actor is the same day as the first day for their spouse (Laurenceau & Bolger, 2011). This distinction allows for the examination of day-specific sources of dependency. To test the associations between actor and partner variables, and to account for the statistical dependence in the data across dyad members, all analyses followed the MIXED procedure in SPSS 20 for repeated measures dyadic data (Kenny, Kashy, & Cook, 2006). This approach analyzes the three levels of data described above as two levels of data, with the lowest level representing multivariate repeated measures (for more information, see Laurenceau & Bolder, 2011). Analyses were run using syntax that combined level 1 and level 2 variables into one equation per model.

In order to keep the analyses theoretically focused and in line with the hypotheses, next day trust was entered as the outcome variable of interest. For each daily diary model predicting trust on day *j*, the predictor variables consisted of the following: the outcome variable on day *j* – 1; actor and partner daily ERS on day *j* – 1; actor and partner attachment anxiety; actor and partner attachment avoidance; and actor and partner symptoms of depression. Main effects for partner variables were included to control for the interdependent nature of dyadic data. The two-way interactions between actor ERS and actor attachment anxiety, actor ERS and actor attachment avoidance, and actor attachment anxiety and avoidance were also included. When examining the influence of partner ERS on trust, the same two-way interactions were included replacing actor ERS with partner ERS (e.g., partner ERS*actor attachment anxiety). In order to examine the influence of gender, a main effect of gender was included, along with the two-way interactions between gender and actor ERS, actor attachment anxiety, and actor attachment avoidance, along with the three-way interactions of gender, actor ERS, and actor attachment anxiety or attachment avoidance. Again, when partner ERS was of interest to the outcome variable (trust), the main and interaction effects of gender included partner ERS rather than actor ERS (e.g., gender*partner ERS*actor attachment anxiety). The unstandardized regression coefficients for each model are presented below. Each model was re-run controlling for relationship length, time cohabitating, and age, separately. Importantly, when controlling for these variables, all significant main and interaction effects remained robust.

## Results

Descriptive statistics by gender for the variables of interest, including the grand mean and standard deviations, are summarized in Table 1. Correlations among key outcome and predictor variables across all individuals are displayed in Table 2.

**Table 1:** Descriptive Statistics for Key Variables by Gender

Variable	Women		Men		*t*
	*M*	*SD*	*M*	*SD*	
ERS	2.50	1.55	1.86	1.02	−13.53**
Attachment anxiety	2.99	1.17	2.65	1.00	1–8.54**
Attachment avoidance	3.05	0.93	3.49	1.07	−12.00**
Depression	9.24	7.84	8.62	7.14	1–2.28**
Daily ERS	1.40	0.89	1.42	0.85	−10.62
Daily negative mood	13.10	4.58	12.99	4.77	1–0.63
Daily trust	6.48	0.97	6.48	1.06	1–0.08

*Note*. ERS = excessive reassurance seeking; *SD* = standard deviation.

**p < .01.

**Table 2:** Correlation Among Key Predictor and Outcome Variables by Gender

Variable	1	2	3	4	5	6	7
1. ERS	.10**	.59**	.13**	.40**	.31**	.43**	−.36**
2. Attachment Anxiety	−.66**	.20**	.43**	.59**	.27**	.29**	−.31**
3. Attachment Avoidance	−.09**	−.25**	.08**	.35**	.07**	.19**	−.22**
4. Depression	−.33**	−.50**	.28**	.29**	.21**	.36**	−.23**
5. Daily ERS	−.16**	−.12**	−.01	−.18**	.19**	.36**	−.22**
6. Daily Negative Mood	−.18**	−.26**	−.09**	−.32**	−.23**	.36**	−.45**
7. Daily Trust	−.12**	−.23**	−.13**	−.38**	−.22**	−.22**	.39**

*Note*. Correlations below the diagonal are for women, while correlations above the diagonal are for men. Correlations between spouses appear along the diagonal. ERS = excessive reassurance seeking.

**p < .01.

### Objective 1: The Contribution of Attachment Style and Trust to Daily ERS

The association between attachment styles and daily ERS, controlling for symptoms of depression, was examined by modeling daily ERS as a function of 1) actor and partner symptoms of depression, 2) actor and partner attachment anxiety and 3) actor and partner attachment avoidance. To examine the influence of gender, a main effect of gender was included along with the two-way interactions between gender and actor symptoms of depression, actor attachment anxiety, and actor attachment avoidance. Analyses were conducted in a hierarchical fashion.

In support of the first hypothesis, individuals high in attachment anxiety engaged in greater daily ERS (*b* = 0.11, *t* = 6.04, *p* < .01). The two-way interaction between gender and actor attachment anxiety was also significant (*b* = −0.08, *t* = −4.69, *p* < .01) such that the association between actor attachment anxiety and daily ERS was stronger for men (*b* = 0.21, *t* = 9.78, *p* < .001) than women (*b* = 0.09, *t* = 4.43, *p* < .001). Consistent with the second hypothesis, individuals high in avoidant attachment reported lower levels of ERS across the 14-day diary period (*b* = −0.05, *t* = −2.82, *p* < .01), with no significant differences between genders (*b* = −0.01, *t* = −0.64, *ns*). An unexpected partner effect also emerged such that individuals with a partner high in attachment anxiety reported higher levels of daily ERS (*b* = 0.07, *t* = 4.12, *p* < .01) whereas individuals with a partner high in attachment avoidance reported engaging in lower levels of daily ERS (*b* = −0.04, *t* = −2.14, *p* < .05).

The association between trust and daily ERS was examined by modeling daily ERS as a function of 1) actor and partner overall trust and 2) relationship length. A main effect of gender was included along with the two-way interactions between gender and overall actor trust and relationship length. Congruent with the third hypothesis, both actor (*b* = −0.06, *t* = −6.69, *p* < .01) and partner trust (*b* = −0.04, *t* = −4.98, *p* < .01) were negatively associated with daily ERS, such that lower levels of trust were associated with more ERS over the 14-day diary period. The two-way interaction between gender and actor trust was also significant (*b* = 0.03, *t* = 3.94, *p* < .01), with the association between actor trust and daily ERS being stronger for men (*b* = −0.09, *t* = −7.19, *p* < .001) than women (*b* = −0.02, *t* = −2.17, *p* < .05). Relationship length was negatively associated with daily ERS (*b* = −0.00, *t* = −2.57, *p* < .05) such that individuals in a longer relationship tended to engage in less daily ERS with no differences between genders.

### Objective 2: The Moderating Properties of Attachment Style on the Relationship Between Daily ERS and Next Day Trust

#### Attachment Style, Daily ERS, and Next Day Trust

**Figure 1:**
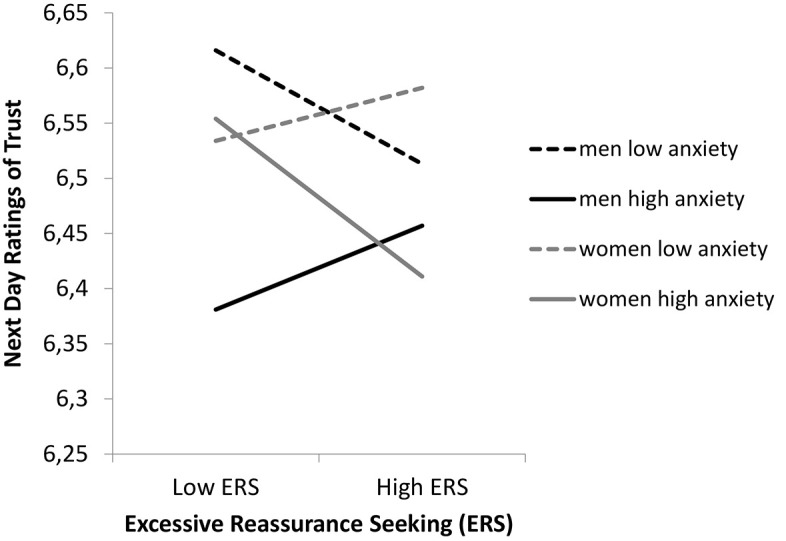
The Moderating Effects of Attachment Anxiety by Gender on the Relationship Between Daily ERS and Next Day Trust

To test the prediction that attachment anxiety, in combination with ERS, would lead to negative interpersonal consequences, the degree to which an individual’s attachment style moderated the relationship between their daily ERS and next day trust was examined. In partial support of the fourth hypothesis attachment anxiety moderated the association between daily ERS and next day trust, but only in women. That is, the three-way interaction between gender, ERS, and actor attachment anxiety was significant (*b* = −0.05, *t* = −2.13, *p* < .05; see Figure 1).

To determine the meaning of the interaction, the simple slopes were examined for both men and women. Regression slopes were computed separately for two values of actors’ attachment anxiety: one standard deviation above the mean and one standard deviation below the mean. For women, there was a negative association between ERS on day *j* − 1 and next day trust, when actors’ attachment anxiety was one standard deviation above the mean (*b* = −0.08, *t* = −2.26, *p* < .05), but not when it was one standard deviation below the mean (*b* = 0.03, *t* = 0.55, *ns*). That is, for women high in attachment anxiety, engaging in ERS was associated with lower levels of next day trust. For men, there was no association between ERS on day *j* − 1 and next day trust at either high (*b* = 0.04, *t* = 0.86, *ns*) or low levels (*b* = −0.06, *t* = −1.06, *ns*) of attachment anxiety.

**Figure 2:**
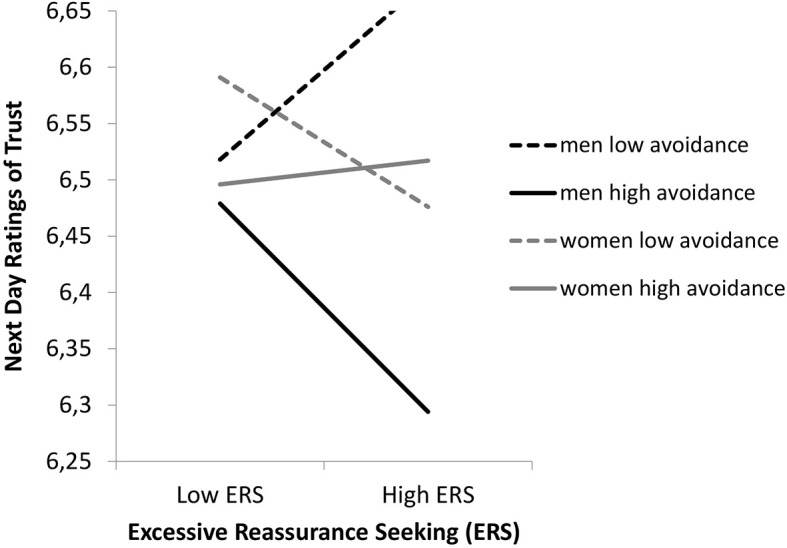
The Moderating Effects of Attachment Avoidance by Gender, on the Relationship Between Daily ERS and Next Day Trust

An unexpected finding was the significant three-way interaction between gender, ERS, and actor attachment avoidance (*b* = 0.07, *t* = 2.90, *p* < .01; see Figure 2).

For women, there was no association between ERS on day *j* − 1 and next day trust at high (*b* = 0.00, *t* = 0.06, *ns*) or low levels (*b* = −0.04, *t* = −1.49, *ns*) of attachment avoidance. For men, there was a negative association between ERS on day *j* − 1 and next day trust, when actors’ attachment avoidance was one standard deviation above the mean (*b* = −0.12, *t* = −3.40, *p* < .01), but not one standard deviation below the mean (*b* = 0.08, *t* = 1.90, *ns*). For men high in attachment avoidance, engaging in ERS was associated with lower levels of trust the following day.

### Objective 3: The Moderating Properties of Actor Attachment Style on the Relationship Between Actor Daily ERS and Partner Next Day Trust

#### Attachment Style, Daily ERS, and Partner Next Day Trust

**Figure 3:**
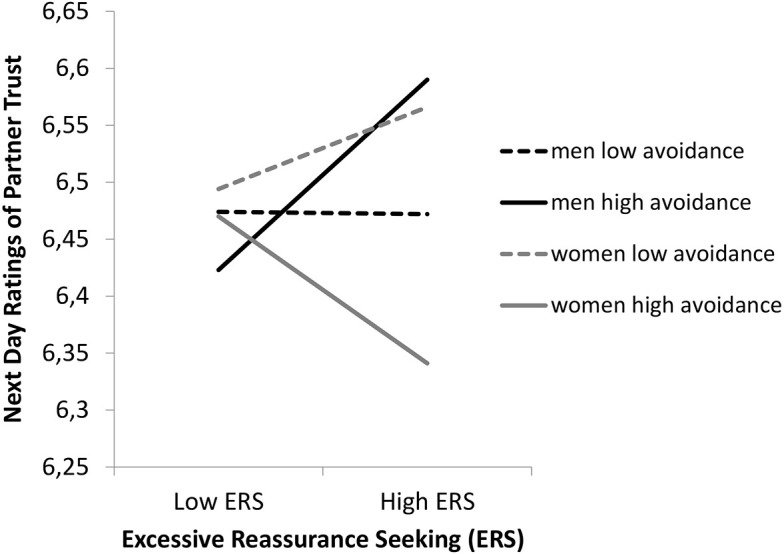
The Moderating Effects of Attachment Avoidance by Gender, on the Relationship Between Daily ERS and Next Day Partner Trust

To test the hypothesis that individuals who are high in attachment anxiety, and engage in ERS, negatively influence their relationship, the degree to which an individual’s attachment style moderated the relationship between their daily ERS and partner next day trust was examined. Support was not found for the fifth hypothesis as the two-way interaction between ERS and attachment anxiety was not significant (*b* = −0.01, *t* = −0.38, *ns*); this finding suggests that for individuals high in attachment anxiety, there was no association between ERS and partner next day trust. An unexpected finding was the significant three-way interaction between gender, ERS, and actor attachment avoidance (*b* = −0.05, *t* = −2.70, *p* < .01; see Figure 3).

For women, there was no significant association between actor ERS on day *j* − 1 and partner next day trust at either high (*b* = −0.07, *t* = −1.74, *ns*) or low levels (*b* = 0.04, *t* = 1.35, *ns*) of attachment avoidance. For men, there was a positive association between actor ERS on day *j* − 1 and partner next day trust, when actors’ attachment avoidance was one standard deviation above the mean (*b* = 0.10, *t* = 2.56, *p* < .05), but not when it was one standard deviation below the mean (*b* = −0.00, *t* = − 0.03, *ns*). That is, for men high in attachment avoidance, engaging in ERS was associated with higher levels of partner reported trust.

### Objective 4: The Moderating Properties of Actor Attachment Style on the Relationship Between Partner Daily ERS and Actor Next Day Trust

#### Attachment Style, Partner Daily ERS, and Next Day Trust

**Figure 4:**
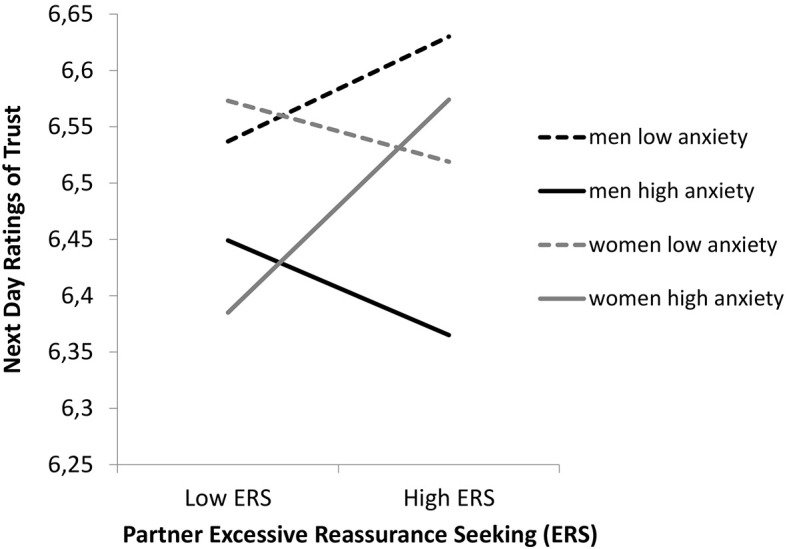
The Moderating Effects of Attachment Anxiety by Gender, on the Relationship Between Daily Partner ERS and Next Day Trust

To test the prediction that individuals with an avoidant attachment style experience negative relational outcomes in response to partner ERS, the degree to which an actor’s attachment style moderated the relationship between their partner’s daily ERS and actor next day trust was examined. The results did not support Hypothesis 6, since the two-way interaction between partner ERS and attachment avoidance was not significant (*b* = −0.03, *t* = −1.55, *ns*). This finding suggests that for individuals with an avoidant attachment style, there was no association between partner ERS and next day trust. An unexpected finding was the three-way interaction between gender, partner ERS, and actor attachment anxiety was significant (*b* = 0.05, *t* = 3.22, *p* < .01; see Figure 4).

For women, there was a positive association between partner ERS on day *j* − 1 and actor next day trust when attachment anxiety was one standard deviation above the mean (*b* = 0.11, *t* = 3.51, *p* < .01), but not when attachment anxiety was one standard deviation below the mean (*b* = −0.03, *t* = −0.69, *ns*). Women high in attachment anxiety with a partner who engaged in greater ERS reported higher levels of next day trust. For men, there was no significant association between partner ERS on day *j* − 1 and actor next day trust at high (*b* = −0.05, *t* = −1.24, *ns*) or low levels (*b* = 0.05, *t* = 1.48, *ns*) of attachment anxiety.

## Discussion

This study provides an original contribution to the literature by affording a comprehensive understanding of how attachment styles influence ERS as it unfolds in real time. In line with prior research, in both men and women, an anxious attachment style was associated with higher levels of daily ERS, and an avoidant attachment style with lower daily ERS. Lower levels of trust were also associated with greater daily ERS. The current study extended the literature by demonstrating that for women high in anxious attachment, engaging in ERS leads to lower levels of trust, rather than decreasing relationship insecurities. Men with an avoidant attachment style also reported lower levels of trust following ERS; in contrast, their partners reported higher levels of trust. This study was also the first to examine how an individual’s attachment style influenced how they perceived ERS from their partner. Women with an anxious attachment style, for example, seemed to appreciate it when their male partners engaged in ERS, as illustrated by higher levels of reported trust following partner ERS. These results support the idea that attachment styles play an important role in determining whether or not ERS will lead to negative interpersonal consequences.

### Objective 1: The Contribution of Attachment Style and Trust to Daily ERS

Part of the first objective was to replicate the positive association found in the literature between attachment anxiety and overall and daily ERS (Abela et al., 2005; Davila, 2001; Evraire & Dozois, 2014; Evraire et al., 2014; Katz et al., 2009; Shaver et al., 2005). In support of prior research and the first hypothesis, higher attachment anxiety was associated with greater levels of daily ERS in both men and women. This finding is the first direct replication of Shaver et al. (2005), who also found that higher levels of attachment anxiety were associated with greater daily ERS from a romantic partner. The current replication offers an important contribution to the literature as it provides the first examination of these associations using a daily diary design with a sample of individuals recruited from the community. Individuals with an anxious attachment style have low self-worth, a fear of abandonment, and use hyperactivating strategies (e.g., proximity seeking) to manage distress (Bartholomew, 1990; Cobb & Davila, 2009; Moran et al., 2008). As such, they likely engage in reassurance seeking with close others, as a means of determining their self-worth and security in their relationships (Brennan & Carnelley, 1999). This finding augments the literature by offering additional support regarding the relationship between an IWM reflecting a high level of concern surrounding relationships, in particular a fear of abandonment or rejection, and ERS.

The second aspect of objective one involved an examination of the association between attachment avoidance and daily ERS. In line with the second hypothesis, higher attachment avoidance was associated with lower levels of daily ERS in both men and women. The association between attachment avoidance and ERS in the literature is mixed, with some studies finding no association between the two variables (Davila, 2001; Shaver et al., 2005) and others finding a negative association (Davila, 2001; Evraire & Dozois, 2014; Evraire et al., 2014). Conceptually, however, it makes sense that individuals with an avoidant attachment style, who tend to minimize the expression of negative emotions and use deactivating strategies to deal with distress, would engage in lower levels of ERS. This study examined the influence of partner attachment style on an individual’s daily ERS. Individuals with a partner high in attachment anxiety reported greater daily ERS, whereas individuals with a partner high in attachment avoidance reported lower levels of daily ERS. These findings are the first to suggest that individuals may tailor their reassurance seeking behavior as a reflection of their partner’s attachment style.

This study was also the first to examine how relationship trust is associated with daily ERS. Consistent with the third hypothesis, lower levels of actor and partner trust were associated with greater daily ERS in both men and women. Trust is defined on the basis of dependability, or the confidence that an individual’s partner will be concerned about and responsive to their needs, desires, and goals, along with faith in the future of the relationship (Mikulincer, 1998). An important component of both anxious attachment and trust concerns relationship security and the ability to rely on one’s partner. Individuals high in attachment anxiety hold a negative sense of trust, attach high importance to negative trust related events, and cope with such events by engaging in security seeking behaviors such as ERS (Mikulincer, 1998; Shaver & Hazan, 1993). As such, it would seem that individuals who reported low levels of trust in their relationships, and those who had partners reporting lower levels of trust, may have engaged in ERS as a means of checking on their status in the relationship and abating their insecurities.

Finally, relationship length was also associated with daily ERS such that individuals who had been together longer reported engaging in less ERS. Again, having been together for a longer period of time could signal to couples that they are secure in their relationship, thus decreasing the need to seek reassurance to assess one’s status in the relationship.

### Objective 2: The Moderating Properties of Attachment Style on the Relationship Between Daily ERS and Relationship Trust

Women high in attachment anxiety who engaged in ERS experienced decreases in levels of trust, supporting Hypothesis 4. This finding provides the first direct evidence for a potential explanation as to why individuals high in attachment anxiety do not seem to benefit from the reassuring feedback provided by their partners and, as a result, seek more reassurance. Individuals with an anxious attachment style rely on feedback from others to determine their self-worth and security in their relationships (Mikulincer & Shaver, 2016). However, if after seeking reassurance they feel even more insecure about their relationships, as evidenced by lower levels of trust, their IWM would remain activated, and their fears of rejection and abandonment would fail to be assuaged leading to further engagement in ERS. It is important to note that this finding only captures one side of the relationship. Perhaps a partner’s reactions to such ERS could influence whether or not individuals high in attachment anxiety experience this decrease in trust following ERS. Future studies need to expand this dynamic examination of the ERS model using other diary studies, or by coding live couple interactions to capture moment to moment delivery of, and reactions to, ERS. Although it was unexpected to find this effect in women but not men, the result makes sense given that the men in this sample had lower levels of attachment anxiety than women, and higher levels of attachment avoidance. Future studies should recruit a sample of men high in attachment anxiety to examine whether or not the effect observed in women could be replicated. Additionally, men higher in attachment avoidance who engaged in ERS experienced decreases in levels of trust. Engaging in ERS may have led to decreased trust since individuals with an avoidant attachment style are uncomfortable being vulnerable in relationships, hold negative models of others (as unresponsive and untrustworthy), and typically use deactivating strategies to deal with distress. However, it is difficult to understand this finding given that it only captures one side of the relationship. It may be the case that how the female partner reacts to ERS coming from a typically avoidant male may influence whether or not he experiences a decrease in trust following ERS.

It was expected that attachment anxiety would predict aspects of the ERS model above and beyond depression. In support of this prediction and previous literature (Evraire & Dozois, 2011, 2014; Evraire et al., 2014), both attachment anxiety and attachment avoidance demonstrated incremental predictive power for ERS over and above the influence of depression. Given these results, the details of Coyne’s model may need to be refined to incorporate these findings.

### Objective 3: The Moderating Properties of Attachment Style on the Relationship Between Daily ERS and Partner Next Day Relationship Trust

It was predicted that the way in which individuals high in attachment anxiety engaged in ERS would negatively impact their partner’s degree of trust; however, this was not supported. It could be the case that partners only react negatively to ERS from their anxiously attached partner after several days of constant reassurance seeking; or significant changes in a partner’s mood, relationship quality, or trust in response to ERS may take a few days to surface. Growth curve analyses would be able to account for such delays. Furthermore, for these analyses, the attachment style of the partner was not accounted for and could influence how they react to receiving ERS from an anxiously attached individual.

A finding that was not predicted was that when men high in attachment avoidance engaged in daily ERS, their partners experienced positive relationship benefits as reflected in increases in next day trust. This finding is in line with Reis and Shaver’s (1988) model which suggests that behavioral support-seeking exchanges between two partners serve as an important foundation for the creation of close bonds. Naturally, individuals with an avoidant attachment style have a tendency to conceal their negative emotions and downplay the importance of receiving support from their partner when coping with distress (Cobb & Davila, 2009; Moran et al., 2008). These dismissive tendencies may lead romantic partners to perceive that their potential contributions to their partner’s coping process are not acknowledged or appreciated (Chow, Buhrmester, & Tan, 2014). As a result, individuals who exhibit an avoidant attachment style tend to perceive their romantic relationships as less intimate, and their partners do the same (Chow et al., 2014). By engaging in ERS, individuals high in attachment avoidance would likely signal to their partners that they are interested in receiving support, which could reflect to their partners a greater sense of closeness. An increase in closeness may also contribute to increases in trust, or the dependability of one’s partner, overall faith in the relationship, and confidence that one’s partner will be an active participant in the relationship (Campbell & Stanton, 2019).

These findings augment the literature and suggest that there may be both secure and insecure forms of reassurance seeking (Evraire & Dozois, 2011, 2014; Girme et al., 2016; Shaver et al., 2005). It may not be the behavior or frequency of ERS per se, but rather characteristics of the individual in combination with reassurance seeking that are associated with negative psychological and/or interpersonal consequences. For example, for individuals high in attachment avoidance, who typically distance themselves from the support of close others, engaging in ERS actually has positive benefits on their relationship. Consistent with this idea, Evraire and Dozois (2014) found that for individuals high in avoidant attachment, there was no association between ERS and symptoms of depression 6 weeks later. Shaver et al. (2005) also found that for highly anxious women, reassurance seeking on a given day was associated with more negative mood the next day; however, for non-anxious women, reassurance seeking on a given day led to positive mood the next day. Furthermore, in previous research examining ERS, including the current study, mean ERS levels are actually quite low (e.g., 1.86 to 3.04 out of 7), further suggesting that reassurance seeking may not be excessive in terms of frequency, but rather how it is delivered as a result of an individual’s IWM of core-beliefs, or symptoms of depression (Evraire & Dozois, 2011).

### Objective 4: The Moderating Properties of Attachment Style on the Relationship Between Partner Daily ERS and Actor Next Day Relationship Trust

Attachment anxiety influenced how women reacted to receiving ERS from their male partners in terms of trust. More specifically, women high in attachment anxiety, who tend to engage in ERS themselves, reacted positively to receiving ERS from their partners by reporting higher levels of trust. Although a novel finding, this result may be explained in part by the similarity hypothesis in partner preference and selection based on attachment styles (for a review, see Holmes & Johnson, 2009; Strauss, Morry, & Kito, 2012). The similarity hypothesis proposes that individuals choose romantic partners who have a similar attachment style as the self, and are more satisfied in their relationships with such partners. Higher levels of attitude similarity between individuals are also related to increased fondness (Miller, Perlman, & Brehm, 2007). Alternative hypotheses to consider with respect to partner selection include the complementarity hypothesis (a preference for partners who fall in the opposite regions of anxiety and avoidance to the self) and the security hypothesis (security is preferred over insecure types of attachment). In a recent test of all three hypotheses, Strauss et al. (2012) examined the relationships between self-attachment style, perceptions of partner attachment style, attachment style of an ideal partner, and relationship variables (satisfaction, trust, supportiveness and feeling validated). Overall, when describing their ideal partner, individuals had a preference for similar but more secure partners (lower anxiety and lower avoidance). Individuals who perceived their current partners as being similar to the self, and greater similarity on attachment anxiety, in particular, predicted positive relationship outcomes including relationship satisfaction and trust. Congruent with the similarity hypothesis, and given that ERS seems to reflect the IWM of an individual with an anxious attachment style (fear of abandonment or rejection), individuals high in attachment anxiety likely interpret partner ERS as an indication of similarity and thus experience increases in trust as a result. In terms of gender differences, again it may be the case that this effect was not observed in men given that they reported lower levels of attachment anxiety and higher levels of attachment avoidance than the women in this sample.

In contrast to our predictions, attachment avoidance did not seem to negatively influence how men or women reacted to partner ERS in terms of trust. This result supports the complementary hypothesis of partner selection, which suggests that individuals prefer partners who fall in the opposite region as them on the dimensions of attachment anxiety and avoidance. Although Strauss et al. (2012) found support for the similarity and security hypotheses with respect to attachment anxiety, higher avoidance predicted ideals and perceptions of the partner that were higher in anxiety (complementary). The complementarity hypothesis predicts that individuals high in attachment avoidance would prefer anxiously attached partners because they confirm their attachment-related expectations that others are dependent and clingy (Holmes & Johnson, 2009). This idea also fits with self-verification theory which proposes that individuals have a strong desire to maintain a predictable social environment by interacting with others who confirm their expectations, allowing for the maintenance of consistent self-image (Swann, 1983; Swann, Stein-Seroussie, & Giesler, 1992).

Although supportive of the complementary hypothesis, the current results are not consistent with Shaver et al. (2005), who found that avoidant women experienced ERS by their male partners as aversive, having a negative influence on relationship quality. One potential explanation for this discrepancy has to do with the difference in relationship characteristics across studies. Shaver et al.’s study included a younger undergraduate sample, none of whom were married, with a median relationship length of 1 year. The current sample consisted of older couples from the community who were all living together, half of whom were married, and had been together for an average length of 8 years. These differences may have been influential given that the literature has found support for the complementarity hypothesis in more long-term relationships, whereas the similarity hypothesis tends to be more characteristic of relationships in the earlier stages (for a review see, Holmes & Johnson, 2009).

### Limitations and Implications

Although the current study contributes to the ERS literature in a number of important ways, its limitations should be noted. The first potential limitation has to do with the fact that attachment anxiety and avoidance were conceptualized as dispositional determinants of ERS and relationship variables. However, some could argue that ERS or relationship qualities could influence the development of an individual’s attachment style. In support of the conceptualization of attachment styles as dispositions, the attachment literature has demonstrated temporal stability in attachment scores over months and even years (Sroufe, 2005; Sroufe, Egeland, Carlson, & Collins, 2005). That being said, if attachment was measured when participants were younger, and reassurance seeking was assessed at the current time point, the idea that insecure attachment can cause certain maladaptive feedback-seeking behaviors may have been supported. As such, future studies should consider replicating the current study using a longitudinal design.

Second, all of the analyses were based on self-report measures of depression, ERS, and trust, rather than interview or behavioral observations. In future research, it would be interesting to study the dynamics of the ERS model explored in the current study as they unfold in real time during couple interactions. Observational methods of ERS, such as those used by Joiner and Metalsky (2001), Knobloch, Knobloch-Fedders, and Durbin (2011), and Stewart and Harkness (2017) could be implemented in such a study

It is important to note that one possible criticism of the current study surrounds the possible conceptual overlap between the constructs of attachment anxiety is trust. That is, it could be argued that a key component of attachment anxiety is doubt or mistrust in a partner’s positive regard and reliable provision of support during times of need, therefore the use of attachment as a predictor and trust as an outcome is redundant. While the two constructs are related, it is worth noting that trust is only one component of attachment, and these two variables are only moderately correlated at best (*r* = −.23 for women and *r* = −.31 for men; as in Table 2).

A final limitation to note concerns the generalizability of the current findings. The demographics of the current sample were relatively homogenous, as the majority of couples were White, highly educated, employed outside of the home, and relatively satisfied with their relationship. As such, the applicability of these findings to different populations of individuals (e.g., couples with poor relationship quality, different ethnicities, unemployed) remains uncertain. In addition, because random sampling was not used in this study, the extent to which the findings from our sample of dyads is fully generalizable to couples in the community is not known.

The findings of the current study have a number of important theoretical and clinical implications. Both attachment anxiety and attachment avoidance showed incremental predictive power for aspects of the ERS model over and above the influence of depression. Thus, the current findings suggest that individuals may not engage in ERS solely because of symptoms of depression, but also as a function of IWM’s reflecting insecurity in relationships. As such, the details of Coyne’s (1976) interpersonal theory of depression may need to be refined to incorporate these findings. The ERS model may also need to be re-conceptualized to account for the notion that there may be both secure and insecure forms of reassurance-seeking, with the insecure form being excessive in that it leads to negative psychological or interpersonal consequences. For example, the results of the current study suggest that it is not the behavior or frequency of ERS per se that is associated with negative relational outcomes; rather, it is the combination of an IWM reflecting insecurity in relationships, and ERS, that leads to negative social consequences. Seeking reassurance actually seems to have beneficial relational effects for secure individuals or those high in the avoidant attachment. The current study was also the first to suggest that ERS may be received differently depending on partner attachment styles, which could in turn moderate the psychological or relational consequences of the reassurance seeking.

Notably, the majority of research in the ERS literature to date has been conducted using undergraduate samples with restricted age ranges and relationship durations. Thus, the current study offers an important contribution to the literature, as the results are based on a sample of community couples reporting a wide range of ages and relationship lengths. This provides support for the generalizability of the extant research demonstrating an association between attachment style, ERS behaviour, and relationship outcomes. Moreover, the current study suggests that the moderating effects of attachment occur regardless of relationship length, suggesting that these observed effects may persist across different stages of romantic relationship development. It is important to note that, although the overall frequency of ERS behaviour appears to decrease across the duration of relationships, to the extent that individuals *do* continue to engage in ERS, the current pattern of findings related to attachment and relationship quality remain the same. This is in line with previous findings in the literature reporting no moderating effects of relationship duration on ERS processes and its negative relational consequences (Shaver et al., 2005).

A number of clinical implications are also evident from these results. It is clear from the findings, as well as previous research, that individuals who engage in ERS do so because of an IWM reflecting a fear of abandonment/rejection or insecurity in relationships. Furthermore, it is clear that although individuals likely engage in ERS to assuage relationship insecurities, ERS seems to decrease levels of trust and likely increase fears about the relationship. As such, clinical interventions targeted towards ERS can focus on this theme with the hopes of helping individuals develop more effective strategies for decreasing their relationship insecurities. Given the dyadic nature of the ERS model, including partners in therapy sessions for individuals who engage in ERS would likely enhance change in the individual as well as improve their social environment. While the reasons behind an individual’s ERS are important targets for treatment, the current study demonstrated that how close others perceive and react to ERS is equally important. Finally, this study emphasized the importance of considering both individuals in a dyad as agents of change and applying this notion to therapy.

### Conclusion

The current study accounted for a number of limitations in the ERS literature and improved upon the lack of methodological diversity in this area of research. It was the first longitudinal daily diary study to account for the dyadic nature of the ERS model, by accounting for the statistical dependence of couples across analyses. Given that the vast majority of past research has examined ERS in undergraduate samples, the current study was the first to investigate this model in a community sample of romantic couples, while also examining how individual and relationship characteristics influenced this process. In line with the ERS literature, an anxious attachment style was associated with higher levels of daily ERS, and an avoidant attachment style with lower ERS. This study was also the first to provide evidence that ERS does not actually work to assuage the relationship insecurities of women high in attachment anxiety. This study contributed novel findings to the literature by demonstrating that attachment styles not only influence the effects of ERS on individual outcome variables, but also influence how an individual perceives and reacts to their partner’s ERS. The results support the idea that there are both insecure and secure forms of reassurance seeking and demonstrated that attachment styles play an important role in determining whether or not ERS will lead to negative interpersonal consequences. Ideally this research highlights the importance of considering both dyads when examining the dynamics of the ERS model and as agents of change in terms of relationship functioning.
